# Decreased preoperative serum 25-Hydroxyvitamin D levels in colorectal cancer are associated with systemic inflammation and serrated morphology

**DOI:** 10.1038/srep36519

**Published:** 2016-11-07

**Authors:** Juha P. Väyrynen, Shivaprakash J. Mutt, Karl-Heinz Herzig, Sara A. Väyrynen, Tiina Kantola, Toni Karhu, Tuomo J. Karttunen, Kai Klintrup, Jyrki Mäkelä, Markus J. Mäkinen, Anne Tuomisto

**Affiliations:** 1 Cancer and Translational Medicine Research Unit, University of Oulu, POB 5000, 90014 Oulu, Finland; 2 Oulu University Hospital and Medical Research Center Oulu, POB 21, 90029 Oulu, Finland; 3 Research Unit of Biomedicine and Biocenter Oulu, University of Oulu, POB 5000, 90014 Oulu, Finland; 4 Department of Gastroenterology and Metabolism, Poznan University of Medical Sciences, 27/33 Szpitalna Str., 60-572, Poland; 5 Research Unit of Surgery, Anesthesia and Intensive Care, University of Oulu, POB 5000, 90014 Oulu, Finland

## Abstract

Deficiency of vitamin D is associated with increased risk of several types of cancer including colorectal cancer (CRC). However, factors contributing to low levels of 25-hydroxyvitamin D [25(OH)D] in CRC are not clear. Therefore, in this study serum 25(OH)D levels in 117 CRC patients and 86 controls were analyzed and correlated with the clinicopathological data including morphological subtype (serrated or conventional), quantity of tumor infiltrating immune cells, levels of systemic inflammatory markers, and disease outcome. We found that the patients had lower serum 25(OH)D levels compared to the controls. Interestingly, among the patients mismatch repair deficiency, serrated morphology, and high body mass index associated with lowest serum 25(OH)D levels. In addition, patients operated in summer or autumn had higher serum 25(OH)D levels. Furthermore, serum 25(OH)D levels inversely correlated with several systemic inflammatory markers, e.g. serum C reactive protein, but did not associate with prognosis. Mechanism leading to vitamin D deficiency in these patients are not clear but could be related to the effects of systemic inflammation. Longitudinal studies are warranted to assess vitamin D deficiency as a potential risk factor for serrated colorectal polyps and adenocarcinoma.

Acquired from the diet or synthesis in the skin under sunlight exposure, vitamin D is hydroxylated in the liver into the major circulating form, 25-hydroxyvitamin D [25(OH)D], which is commonly used to determine the patients’ vitamin D status^1^,^2^. There is no universally accepted definition of the normal range of human 25(OH)D levels, but levels of 30–100 ng/ml (75 to 250 nmol/L) are considered to fall within the normal limits and levels of 0 to 20 ng/ml (0–50 nmol/L) are considered deficient. The hydroxylation of 25(OH)D into the hormonally active form of vitamin D (1,25(OH)_2_D_3_) takes place in the kidneys and also in most extrarenal tissues, where it acts in a paracrine manner^1^. The hormonally active form, 1,25(OH)_2_D_3_, has a short half-life and tight homeostatic control^1^. The classical role of vitamin D is to regulate mineral homeostasis and to control bone metabolism, while other functions include the regulation of immune responses, the induction of cell differentiation, the stimulation of apoptosis, and the inhibition of cell proliferation, angiogenesis, and metastasis^3^,^4^,^5^.

Colorectal cancer (CRC) is the second most common fatal malignancy in the Western world. CRC is a multi-pathway disease, and 10–30% of the cases are considered to develop from the serrated colorectal polyps and evolve along the serrated pathway^6^. Serrated colorectal adenocarcinoma (SAC) can be distinguished by its characteristic morphology^6^, which reflects its unique messenger RNA expression profile compared to conventional colorectal adenocarcinoma (CC)^7^.

Vitamin D deficiency has been associated with a variety of cancers^1^,^3^, and epidemiological studies have also demonstrated an association between vitamin D deficiency and an increased risk of colorectal cancer (CRC)^8^,^9^. Moreover, low plasma prediagnostic^10^ and postoperative^11^ 25(OH)D levels in CRC patients have been associated with adverse prognosis, according to meta-analyses^12^,^13^. However, the determinants of preoperative 25(OH)D levels are incompletely known^14^. Although vitamin D has been linked with an anti-inflammatory function^3^, the associations between preoperative serum 25(OH)D levels in CRC and tumor associated immune/inflammatory cell reaction or systemic levels of the inflammatory mediators and markers have not been well-characterized. Finally, there is no information about the association between serum 25(OH)D levels and different pathways of CRC development.

In this study, we have analyzed the preoperative serum 25(OH)D levels in a series of 117 prospectively recruited CRC patients and 86 healthy matched controls in Northern Finland (latitude 65° North). Especially, the aim was to characterize the association of serum 25(OH)D levels with the developmental route, with details of local and systemic inflammatory reaction patterns, and with survival.

## Results

### Serum 25(OH)D in CRC patients and healthy controls

There were no significant differences in the average age or sex distribution between the CRC patients and the controls (Table S1). The median body mass index (BMI) of the patients was 26.3, while no data on BMI was available for controls aged less than 65 (healthy blood donor group). However, there was no significant difference in the BMI of the patients aged 65 or more compared to the respective controls (median 26.6 vs. 26.9, p = 0.205). The patients had significantly lower serum 25(OH)D levels relative to the controls (median 49.0 nmol/L vs. 59.5 nmol/L, p = 6.6E-5). Receiver operating characteristics (ROC) analysis indicated an area under the curve (AUC) of 0.662 (95% CI 0.59-0.74) for serum 25(OH)D in the discrimination of the cases and controls, and using a cut-off of 50 nmol/L, the sensitivity was 80.2% and the specificity was 52.1%

### Serum 25(OH)D levels and clinical and pathological characteristics

Serum 25(OH)D levels did not significantly correlate with patient age (p = 0.746) or gender (p = 0.204), tumor location (p = 0.116), TNM stage (p = 0.420), and WHO grade (p = 0.205) (Table 1). However, the patients with body BMI > 30 had lower serum 25(OH)D levels relative to those with BMI ≤ 30 (p = 0.0032), and also the patients operated in winter or spring had lower serum 25(OH)D levels (p = 0.012). SAC associated with decreased serum 25(OH)D levels relative to the CC (p = 0.029). Mismatch repair (MMR) deficiency is characteristic to Lynch syndrome (hereditary nonpolyposis colorectal cancer) and frequent in the serrated route of CRC^6^, and was associated with reduced serum 25(OH)D levels (p = 0.018). However, the presence of BRAF or KRAS mutation, also most frequently observed in SAC^6^, did not significantly correlate with serum 25(OH)D levels (p = 0.512).

### Serum 25(OH)D, immune cell infiltration, and systemic inflammatory markers

To evaluate the potential effects of the immune-modulating functions of Vitamin D in CRC, we analyzed the associations between serum 25(OH)D levels and systemic inflammatory markers (Table 2), as well as local inflammatory cell densities in CRC tissue (Table S2). Serum 25(OH)D levels inversely correlated with an assemblage of systemic inflammatory markers, most notably with blood neutrophil count (p = 0.0012), serum C-reactive protein (CRP) levels (p = 0.0021), blood neutrophil/lymphocyte ratio (NLR) (p = 0.0041), and serum interleukin (IL)-6 levels (p = 0.0042) (Table 2). Instead, of the studied types of tumor infiltrating immune cells, only intratumoral CD1a^+^ dendritic cells (p = 0.012) and neutrophils (p = 0.027) showed significant correlation with serum 25(OH)D levels (Table S2).

### Multiple linear regression models

Multiple linear regression modeling was utilized to evaluate the independent significance of different explanatory variables on serum 25(OH)D levels (Table 3). The models indicated that sunlight exposure, *i.e.* serum samples taken during winter or spring, serrated histology, and blood neutrophil count were independent predictors of low serum 25(OH)D levels.

### Serum 25(OH)D and survival

**Figure 1:**
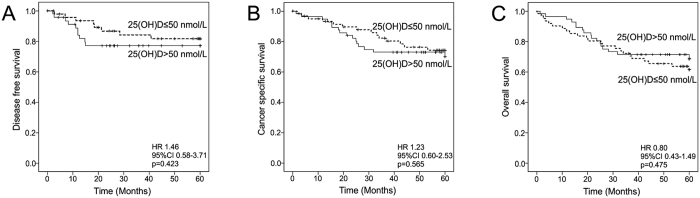
Kaplan-Meier curves demonstrating the associations between serum 25(OH)D levels and disease-free survival (DFS), cancer-specific survival (CSS), and overall survival (OS). (**A**) Serum 25(OH)D and DFS. (**B**) Serum 25(OH)D and CSS. (**C**) Serum 25(OH)D and OS. Abbreviations: CI: confidence interval; HR: hazard ratio.

Finally, we evaluated the association between serum 25(OH)D levels and disease outcome. ROC analysis indicated an AUC of 0.567 (95% CI 0.419–0.716) for the detection of patients with recurrent disease in 60 month follow-up. Further analyses utilizing different cut-of points ranging from 30 to 70 nmol/L indicated that serum 25(OH)D did not significantly associate with disease-free, cancer-specific, or overall survival in 60 month follow-up (Table S3). Kaplan-Meier curves with a cut-off point of 50 nmol/L are presented as Fig. 1.

## Discussion

In recent decades, the deficiency of vitamin D has been implicated in several chronic metabolic, cardiovascular, and neoplastic diseases^3^. The present study investigated the factors contributing to preoperative serum 25(OH)D levels in CRC and their significance. According to our observations, the development of cancer by the serrated pathway, systemic inflammation, and obesity are associated with vitamin D deficiency in CRC.

Our study indicates that CRC patients have lower serum 25(OH)D levels as compared to healthy controls. Earlier studies have associated low serum 25(OH)D levels with an increased risk of developing CRC^8^,^9^, while the results of the studies assessing preoperative values in cancer patients have been contradictory^15^,^16^. Interestingly, the median serum 25(OH)D levels in stage I patients did not differ from the levels of healthy controls, but low median serum 25(OH)D levels were associated to the stage II-IV CRC. This finding suggests that the decrease in serum 25(OH)D may be related to the progression of CRC, also supported by some earlier results^15^.

The progression of cancer is associated with the activation of systemic inflammatory response that may regulate and promote metastasis^17^. In CRC, systemic inflammatory response, as evidenced by increased serum CRP and decreased serum albumin (mGPS) or increased blood neutrophil/lymphocyte ratio, indicates adverse prognosis^17^. We report here of an association between low serum 25(OH)D levels and high scores of systemic inflammatory response in CRC. Similar association has been found in healthy adults and general patient material^18^. This association may reflect the immunomodulatory and immunosuppressive functions of vitamin D^19^, proinflammatory cytokines suppressing hepatic production of carrier proteins of vitamin D, or redistribution or consumption of vitamin D storages by the systemic inflammatory response^18^. The results support the suggestion by Conway and McMillan^20^ that some of the studies addressing the association between circulating 25(OH)D concentrations and CRC outcome may have been confounded by the effect of systemic inflammatory response. Indeed, also in the multivariate analysis, the systemic inflammatory response, as indicated by high blood neutrophil count, had a higher association with low serum 25(OH)D levels than high BMI.

Immune cell infiltration has frequently been associated with improved survival in CRC^21^,^22^. It was recently proposed that prediagnostic vitamin D deficiency is a risk factor for CRC with intense intratumoral periglandular immune reaction^23^. Our analyses indicate that serum 25(OH)D concentrations positively correlate with the densities of CD1a^+^ dendritic cells and neutrophils at the tumor stroma but not with T cells that are considered more important in tumor immunosurveillance and have better-established prognostic value^21^,^22^. The mechanism and the significance of the association we observed is not clear, but may be related to the well accepted immunoregulatory role of 25(OH)D on dendritic cells^24^ and requires further investigation. However, our findings do not suggest that tumor inflammatory infiltrate, in overall, is an important determinant of serum 25(OH)D levels in CRC patients or vice versa.

The patients with tumors showing serrated histology had lower serum 25(OH)D levels as compared with those with non-serrated histology. Similar association was seen with MMR deficiency, a characteristic feature of SAC. These associations suggest that vitamin D deficiency might be related to the serrated pathway of CRC. Earlier studies have reported that vitamin D deficiency is not a risk factor for colorectal hyperplastic polyps^25^, but to our knowledge, little is known of the association between vitamin D deficiency and sessile and traditional serrated adenomas, which is an important subject for further investigation, since hyperplastic polyps are very common, and the risk of neoplastic progression has not been attributed to hyperplastic polyps but sessile and traditional serrated adenomas^6^.

Our data indicates an inverse correlation between serum 25(OH)D levels and BMI in CRC patients, and earlier studies support similar association in healthy adults^26^,^27^. The subcutaneous fat may store more vitamin D synthesized in the skin, and obese persons have significantly lower increase in serum 25(OH)D after UV-B exposure^26^. Moreover, obesity is associated with low level of physical activity, which may increase the time spend inside, thus decreasing UV exposure and serum 25(OH)D levels. As vitamin D deficiency, obesity is also associated with increased risk of CRC, and it has been suggested that vitamin D deficiency in obese people may explain at least 20% of cancer risk attributable to high BMI^28^.

There is variation in circulating 25(OH)D in Finnish population due to the seasonal changes in the exposure to sunlight and low vitamin D intake^29^,^30^. Hassi *et al.*^31^ have evaluated the luminosity in our latitude and only from June to October, the luminosity is sufficient for Vitamin D production in the skin. Accordingly, our results indicate that patients operated in winter or spring have significantly higher likelihood of low serum 25(OH)D, and the season of operation was the most important predictor of 25(OH)D in the multivariate analyses. This result points out the importance to take this confounding factor into account also in the subsequent studies. Whether this seasonal variation in serum levels of 25(OH)D has clinical significance in cancer patients, is not well-known. This study did not include any dietary surveys but previous studies have reported that the intake of vitamin D in the area is most frequently below the recommended intake^32^. A previously published dietary survey of 8960 subjects born in 1966 in the Northern Finland indicated that only roughly one fourth had regular fish intake^33^. Moreover, in a Nordic multi-center trial with subjects (n = 213) with metabolic syndrome randomized to a control or a healthy Nordic diet favoring fish (≥300 g/week, including ≥200 g/week fatty fish) among other healthy Nordic products^34^, the healthy Nordic diet intervention increased vitamin D intake but not plasma 25(OH)D concentration. The reason, why fish consumption did not improve vitamin D status might be that many fish are farmed and might contain little vitamin D or that frying fish may result in vitamin D destruction^35^. All in all, the lack of dietary surveys is a limitation of this study, which could cause confounding of the results, since for example, the use of vitamin D supplements could increase serum 25(OH)D levels.

There are also other limitations have to be taken account, when interpreting the results of the study. First, no surveys on physical activity were conducted, and we cannot rule out the effect of physical activity. However, we did not observe any difference between serum 25(OH)D levels in stage II-IV patients (Table 1). All patients participating this study were eligible to surgery. Therefore it is unlikely that physical inactivity due to disease burden would play a major role in explaining decreased serum 25(OH)D levels. Second, the BMI is another potential confounding factor, and no BMI data was available for controls aged less than 65. Nevertheless, the BMI of the patients aged 65 or more did not differ from that of the respective controls, and also within the CRC patient subgroup, the multivariate analysis indicated that the associations between lower serum 25(OH)D levels and systemic inflammation and serrated histology were independent of the BMI of the patients. The advantages of the study included a prospectively recruited, well-characterized study population including patients from different stages and with a plethora of analyzed markers of systemic and local inflammatory response. The patients were from a single surgical unit and had uniform follow-up schedule.

In conclusion, decreased serum 25(OH)D levels in CRC are associated with serrated tumor morphology, and systemic inflammatory response. Further experimental studies are warranted to address biological mechanisms underlying the findings and possible role of low vitamin D levels in the development of serrated CRC.

## Patients and Methods

### Patients and controls

All newly diagnosed CRC patients operated in Oulu University Hospital, between April 2006 and January 2010 (n = 344) were introduced for this prospective study. The study design was approved by the Ethical Committee of Oulu University Hospital (58/2005, 184/2009), and the methods were performed in accordance with the relevant guidelines and regulations. Preoperative blood samples and surgical specimens were originally collected from 149 patients, who had signed an informed consent to participate and were eligible to the study^36^. 32 of 149 patients (21.5%) received preoperative radiotherapy or chemoradiotherapy (RT/CRT) and were excluded from the analyses since RT/CRT is a potential confounding factor that may affect the histological characteristics of the tumors^37^ and associates with vitamin D deficiency^38^. Patients were followed up in regular intervals for up to five years^39^,^40^. The REporting recommendations for tumor MARKer prognostic studies (REMARK) were taken into account in the study design and reporting^41^.

Age and sex matched control serum samples were acquired from healthy voluntary blood donors (Finnish Red Cross, Oulu, Finland; n = 36, age < 65 years) and cataract surgery patients (Oulu University Hospital; n = 50, age ≥ 65 years). Blood samples from patients and controls were collected and centrifuged, and the serum was stored at −70 °C until further analysis. The study set-up is previously described^36^. The data of the BMI of the patients and the controls aged ≥ 65 years was collected from the clinical records, while no such data was available for controls aged < 65 years^36^.

### Determination of serum 25(OH)D levels and systemic inflammation

Serum 25(OH)D levels were measured using 25-Hydroxy vitamin D enzyme immunoassay (EIA) kit (Immunodiagnostic Systems GmbH, Germany) following manufacturer’s instructions and described previously^42^. For accuracy assessment, each measurement was included with certified materials from National Institute of Standards and Technology, United States (NIST, USA); Standard Reference Material-972 (SRM-972; consists four vials from level 1 to 4) and two internal plasma controls^43^. The inter-assay variations (CVs) ranged within (8.6 to 14.6%) for different control and certified materials. All the measurements were performed blinded to the clinical data.

Differential leukocyte count, serum CRP, and serum albumin was analyzed in the laboratory of Oulu University hospital^36^,^44^, and mGPS was calculated from CRP and albumin values^36^. Concentrations of serum levels of thirteen cytokines were measured as described earlier^36^.

### Histopathological analyses of the tumors and associated inflammatory and immune cell reaction and determination of KRAS and BRAF mutations

Tumors were classified according to TNM6 classification^45^ and their differentiation evaluated according to the WHO criteria^46^. Colorectal cancer associated lymphoid reaction (CLR), denoting lymphoid follicles surrounding the tumors, was assessed as “the number of lymphoid follicles/the length of the invasive front of the tumor” as described earlier^47^. Tumors were classified into SACs and CCs by the established criteria, as described earlier^46^,^48^.

MMR enzyme status was evaluated utilizing MLH1 and MSH2 immunohistochemistry^44^. Utilizing a tissue microarray with one to four (median three) cores of 3.0 mm diameter per case from the invasive margin (IM) and the center of the tumor (CT)^49^, the densities of the immune cell infiltrate at the IM, and the CT (stroma, CT-S; intraepithelial, CT-IEL) were analyzed using ImageJ, a free image analysis software, and a computer assisted counting method as described earlier^49^,^50^. BRAF and KRAS mutation analysis was carried out as described earlier^48^.

### Statistical analyses

Normally distributed continuous variables are presented as mean (standard deviation, SD), whereas other continuous variables are presented as median (interquartile range, IQR). IBM SPSS Statistics for Windows version 22.0 (IBM Corp. Armonk, NY) was used for the statistical analyses. Statistical significances of the associations between serum 25(OH)D levels and categorical variables were analyzed by Mann-Whitney U test (comparing two classes) or Kruskal-Wallis test (comparing three or more classes). Pearson correlation coefficients (r) were used to assess the correlations between two continuous variables. Logarithmic transformation was applied to variables with positive skewness. Multiple linear regression analysis was used to model the relationship between serum 25(OH)D levels and several explanatory variables. Kaplan-Meier method and Log rank test were utilized in the survival analyses. A two-tailed p < 0.05 was considered statistically significant.

## Additional Information

**How to cite this article**: Väyrynen, J. P. *et al.* Decreased preoperative serum 25-Hydroxyvitamin D levels in colorectal cancer are associated with systemic inflammation and serrated morphology. *Sci. Rep.* **6**, 36519; doi: 10.1038/srep36519 (2016).

**Publisher’s note:** Springer Nature remains neutral with regard to jurisdictional claims in published maps and institutional affiliations.

## Supplementary Material

Supplementary Information

## Figures and Tables

**Table 1:** Serum 25(OH)D levels in colorectal cancer patients in relation to clinical and pathological characteristics.

	serum 25(OH)D, nmol/L, median (IQR)	p value
Gender		
Male (n = 58)	52.7 (38.3–69.5)	0.204
Female (n = 59)	47.6 (37.7–58.5)	
Age		
<65 years (n = 43)	50.2 (37.7–60.8)	0.746
≥65 years (n = 74)	48.6 (38.9–66.5)	
Body mass index		
<25 (n = 47)	50.4 (37.4–67.0)	0.0032
25–30 (n = 46)	52.7 (45.2–70.5)	
>30 (n = 22)	39.8 (31.7–46.4)	
Time of operation		
Winter (Dec-Feb) (n = 21)	43.6 (30.2–58.1)	0.012
Spring (Mar-May) (n = 34)	42.3 (34.2–54.6)	
Summer (Jun-Aug) (n = 41)	53.6 (46.1–70.7)	
Autumn (Sep-Nov) (n = 21)	52.6 (45.1–63.9)	
Tumor location		
Proximal colon (n = 49)	46.1 (34.9–54.5)	0.116
Distal colon (n = 28)	55.9 (43.9–66.3)	
Rectum (n = 40)	51.6 (39.7–71.0)	
Morphology		
Conventional (n = 89)	52.6 (40.5–65.1)	0.029
Serrated (n = 28)	42.9 (31.0–52.7)	
TNM Stage		
Stage I (n = 19)	60.8 (44.7–75.3)	0.420^A^
Stage II (n = 46)	48.1 (37.4–56.0)	
Stage III (n = 32)	48.1 (38.9–71.7)	
Stage IV (n = 18)	49.8 (31.0–60.3)	
Depth of invasion		
T1 (n = 5)	45.3 (38.3–78.7)	0.267
T2 (n = 19)	60.8 (41.3–76.9)	
T3 (n = 83)	47.9 (37.1–57.8)	
T4 (n = 9)	60.2 (41.1–69.2)	
Nodal metastasis		
N0 (n = 69)	50.0 (38.7–64.2)	0.939
N1 (n = 27)	48.8 (35.8–71.3)	
N2 (n = 19)	50.6 (41.3–59.5)	
Distant metastasis		
M0 (n = 98)	49.0 (39.1–66.5)	0.513
M1 (n = 18)	49.8 (31.0–60.3)	
WHO Grade 1–3		
Grade 1 (n = 16)	56.0 (37.0–71.0)	0.205
Grade 2 (n = 86)	50.3 (39.1–62.1)	
Grade 3 (n = 14)	43.1 (33.6–55.8)	
Mismatch repair (MMR) enzyme status		
MMR-deficient (n = 11)	35.3 (32.0–45.5)	0.018
MMR-proficient (n = 105)	50.4 (39.9–66.2)	
BRAF or KRAS mutation		
BRAF mutation (n = 12)	42.7 (35.3–62.2)	0.512
KRAS mutation (n = 30)	47.2 (37.1–67.4)	
No BRAF nor KRAS mutation (n = 74)	52.6 (39.7–64.1)	
Modified Glasgow Prognostic score (mGPS)		
0 (n = 91)	50.6 (39.2–69.4)	0.050
1–2 (n = 26)	46.1 (33.8–53.9)	

Abbreviations: IQR: interquartile range. The p-values are for Mann-Whitney or Kruskal-Wallis test.

^A^Stage I vs. II-IV: p = 0.119.

**Table 2:** Correlations between serum 25(OH)D and systemic inflammatory markers.

	serum 25(OH)D	
	Pearson r	p value
Serum C-reactive protein	**−0.282**	**0.0021**
Blood Leukocyte count	**−0.234**	**0.011**
Blood Neutrophil count	**−0.298**	**0.0012**
Blood Lymphocyte count	0.058	0.535
Blood Neutrophil/lymphocyte ratio	**−0.264**	**0.0041**
Serum IL-1ra	**−0.195**	**0.035**
Serum IL-4	−0.037	0.688
Serum IL-6	**−0.264**	**0.0042**
Serum IL-7	−0.094	0.311
Serum IL-8	−0.130	0.162
Serum IL-9	−0.030	0.746
Serum IL-12(p70)	−0.081	0.383
Serum IFNγ	−0.036	0.697
Serum CXCL10	−0.037	0.694
Serum CCL2	0.020	0.831
Serum CCL4	0.030	0.744
Serum CCL11	0.163	0.079
Serum PDGF-BB	−0.146	0.116

Numbers indicate Pearson correlation coefficients (r) for logarithmically transformed variables.

**Table 3:** Two multiple linear regression models of the determinants of serum 25(OH)D levels in CRC patients.

Independent	Beta	p value
Model 1 (n = 115; R = 0.394, R^2^ = 0.155)		
Age	0.157	0.082
Measurement in summer or autumn (no vs. yes)	0.311	6.8E-4
Body mass index	−0.106	0.234
Serrated morphology (no vs. yes)	−0.175	0.049
Model 2 (n = 115; R = 0.450, R^2^ = 0.202)		
Age	0.142	0.106
Measurement in summer or autumn (no vs. yes)	0.266	0.0028
Serrated morphology (no vs. yes)	−0.178	0.039
Blood neutrophil count	−0.246	0.0051

Model 1 included patient age as a basic clinical variable and three other clinicopathological variables with the highest statistical significance in the univariate analyses (BMI, season of measurement, and serrated histology). Model 2 was constructed to test, whether also systemic inflammatory markers had independent predictive value on serum 25(OH)D levels. Serum 25(OH)D, blood neutrophil count, and body mass index were logarithmically transformed because of positive skewness.
