# Supplementary material for: Decreased preoperative serum 25-Hydroxyvitamin D levels in colorectal cancer are associated with systemic inflammation and serrated morphology
Source: Sci Rep. 2016 Nov 7;6:36519. doi: 10.1038/srep36519 (PMC5098144; doi:10.1038/srep36519)
Supplement: Supplementary Information [file srep36519-s1.doc]

# Decreased preoperative serum 25-Hydroxyvitamin D levels in colorectal cancer are associated with systemic inflammation and serrated morphology

Juha P. Väyrynen^1,2^, Shivaprakash J. Mutt^3^, Karl-Heinz Herzig^3,4^, Sara A. Väyrynen^1,2^, Tiina Kantola^1,2^, Toni Karhu^3^, Tuomo J. Karttunen^1,2^, Kai Klintrup^2,5^, Jyrki Mäkelä^2,5^, Markus J. Mäkinen^1,2^, Anne Tuomisto^1,2^

**Table S1**. Characteristics of the patients with colorectal carcinoma and the controls.

|  | CRC patients (n=117) | Healthy controls (n = 86) |
| --- | --- | --- |
| Age, mean (SD) | 67.7 (11.2) | 67.2 (10.4) |
| Gender |  |  |
| Male | 58 (49.6%) | 45 (52.3%) |
| Female | 59 (50.4%) | 41 (47.7%) |
| Tumor location |  |  |
| Proximal colon | 49 (41.9%) |  |
| Distal colon | 28 (23.9%) |  |
| Rectum | 40 (34.2%) |  |
| WHO grade |  |  |
| Grade 1 | 16 (13.8%) |  |
| Grade 2 | 86 (74.1%) |  |
| Grade 3 | 14 (12.1%) |  |
| TNM stage |  |  |
| Stage I | 19 (16.5%) |  |
| Stage II | 46 (40.0%) |  |
| Stage III | 32 (27.8%) |  |
| Stage IV | 18 (15.7%) |  |
| Serum 25(OH)D, median (IQR), nmol/L^A^ | 49.0 (38.1–64.2) | 59.5 (51.4–66.7) |
| Body mass index (BMI)^B^ | 26.3 (23.5-28.9) | B |
| Body mass index (BMI) in patients and controls aged >65^C^ | 26.6 (23.7-28.5) | 26.9 (24.6-30.2) |

^A^CRC patients vs. healthy controls; p= 6.6E-5 (Mann-Whitney test). ^B^Data not available for controls aged less than 65 years. ^C^p= 0.205 (Mann-Whitney test). Abbreviations: CRC: colorectal cancer; IQR, interquartile range); SD: standard deviation.

**Table S2**. Correlation between serum vitamin D levels and local areal density of inflammatory cells in CRC specimens.

|  | Serum 25(OH)D | |
| --- | --- | --- |
|  | Pearson r | p value |
| CD3 IM | -0.100 | 0.281 |
| CD3 CT-S | -0.017 | 0.856 |
| CD3 CT-IEL | -0.056 | 0.554 |
| CD8 IM | -0.073 | 0.435 |
| CD8 CT-S | -0.151 | 0.104 |
| CD8 CT-IEL | -0.078 | 0.421 |
| FoxP3 IM | 0.042 | 0.650 |
| FoxP3 CT-S | 0.053 | 0.573 |
| CD68 IM | 0.069 | 0.460 |
| CD68 CT-S | 0.135 | 0.148 |
| CD83 IM | 0.161 | 0.088 |
| CD83 CT-S | -0.105 | 0.270 |
| CD1a IM | 0.068 | 0.464 |
| CD1a CT-S | **0.233** | **0.012** |
| Mast cell tryptase IM | -0.061 | 0.515 |
| Mast cell tryptase CT-S | 0.061 | 0.515 |
| Neutrophil elastase IM | 0.116 | 0.214 |
| Neutrophil elastase CT-S | **0.204** | **0.027** |
| CLR density | -0.086 | 0.359 |

Numbers indicate Pearson correlation coefficients (r) for logarithmically transformed variables. Abbreviations: IM: invasive margin; CT-S, center of the tumor, stroma; CT-IEL, center of the tumor, intraepithelial; CLR: colorectal cancer associated lymphoid reaction.

**Table S3**. Analysis of 60 month disease-free survival (DFS), cancer-specific survival (CSS), and overall survival (OS) according to the serum 25(OH)D levels with different cut-off points

|  | **DFS**^A^ | | | **CSS**^B^ | | | **OS**^C^ | | |
| --- | --- | --- | --- | --- | --- | --- | --- | --- | --- |
|  | HR | 95% CI | p value | HR | 95% CI | p value | HR | 95% CI | p value |
| 25(OH)D (≤30 vs. >30 nmol/L) | 1.58 | 0.21-11.9 | 0.655 | 0.69 | 0.24-1.96 | 0.482 | 0.50 | 0.22-1.13 | 0.096 |
| 25(OH)D (≤50 vs. >50 nmol/L) | 1.46 | 0.58-3.71 | 0.423 | 1.23 | 0.60-2.53 | 0.565 | 0.80 | 0.43-1.49 | 0.475 |
| 25(OH)D (≤70 vs. >70 nmol/L) | 1.99 | 0.75-5.31 | 0.168 | 0.99 | 0.41-2.43 | 0.987 | 0.70 | 0.29-1.67 | 0.425 |

^A^n=95; median follow-up time 53.0 months (IQR 20.1-60.0); 18 (18.9%) events; 22 (18.8%) cases excluded from the analysis because the operation was not radical

^B^n=117; median follow-up time 57.9 months (IQR 29.5-60.0); 30 (25.6%) events

^C^n=117; median follow-up time 57.9 months (IQR 29.5-60.0); 40 (34.2%) events
